# Multiplex genomic profiling of non–small cell lung cancers from the LETS phase III trial of first-line S-1/carboplatin versus paclitaxel/carboplatin: results of a West Japan Oncology Group study

**DOI:** 10.18632/oncotarget.1906

**Published:** 2014-04-17

**Authors:** Isamu Okamoto, Kazuko Sakai, Satoshi Morita, Hiroshige Yoshioka, Hiroyasu Kaneda, Koji Takeda, Tomonori Hirashima, Yoshihito Kogure, Tatsuo Kimura, Toshiaki Takahashi, Shinji Atagi, Takashi Seto, Toshiyuki Sawa, Masashi Yamamoto, Miyako Satouchi, Motoyasu Okuno, Seisuke Nagase, Koichi Takayama, Keisuke Tomii, Tadashi Maeda, Satoshi Oizumi, Shinji Fujii, Yusaku Akashi, Kazumi Nishino, Noriyuki Ebi, Kazuhiko Nakagawa, Yoichi Nakanishi, Kazuto Nishio

**Affiliations:** ^1^ Center for Clinical and Translational Research, Kyushu University Hospital, Fukuoka, Japan;; ^2^ Department of Genome Biology, Kinki University Faculty of Medicine, Osaka, Japan;; ^3^ Department of Biomedical Statistics and Bioinformatics, Kyoto University Graduate School of Medicine, Kyoto, Japan;; ^4^ Department of Respiratory Medicine, Kurashiki Central Hospital, Kurashiki, Japan;; ^5^ Department of Medical Oncology, Kinki University Faculty of Medicine, Osaka, Japan;; ^6^ Department of Clinical Oncology, Osaka City General Hospital, Osaka, Japan;; ^7^ Department of Thoracic Malignancy, Osaka Prefectural Medical Center for Respiratory and Allergic Diseases, Osaka, Japan;; ^8^ Department of Respiratory Medicine, National Hospital Organization, Nagoya Medical Center, Nagoya, Japan;; ^9^ Department of Respiratory Medicine, Osaka City University Medical School, Osaka, Japan;; ^10^ Division of Thoracic Oncology, Shizuoka Cancer Center, Nagaizumi, Japan;; ^11^ Department of Respiratory Medicine, National Hospital Organization, Kinki-chuo Chest Medical Center, Osaka, Japan;; ^12^ Department of Thoracic Oncology, National Kyushu Cancer Center, Fukuoka, Japan;; ^13^ Department of Respiratory Medicine and Oncology, Gifu Municipal Hospital, Gifu, Japan;; ^14^ Department of Respiratory Medicine, Nagoya Ekisaikai Hospital, Nagoya, Japan;; ^15^ Department of Thoracic Oncology, Hyogo Cancer Center, Akashi, Japan;; ^16^ Department of Respiratory Medicine, Aichi Cancer Center Aichi Hospital, Okazaki, Japan;; ^17^ Department of Thoracic Surgery, Tokyo Medical University, Tokyo, Japan;; ^18^ Research Institute for Diseases of the Chest, Graduate School of Medical Sciences, Kyushu University, Fukuoka, Japan;; ^19^ Department of Respiratory Medicine, Kobe City Medical Center General Hospital, Kobe, Japan;; ^20^ Department of Medical Oncology, National Hospital Organization Yamaguchi-Ube Medical Center, Ube, Japan;; ^21^ First Department of Medicine, Hokkaido University School of Medicine, Sapporo, Japan;; ^22^ Division of Respiratory Disease, Kumamoto Regional Medical Center, Kumamoto, Japan;; ^23^ Department of Medical Oncology, Nara Hospital Kinki University Faculty of Medicine, Nara, Japan;; ^24^ Department of Thoracic Oncology, Osaka Medical Center for Cancer and Cardiovascular Diseases, Osaka, Japan;; ^25^ Department of Respiratory Oncology, Iizuka Hospital, Fukuoka, Japan

**Keywords:** non–small cell lung cancer, phase III trial, genotyping, fusion gene, MET amplification

## Abstract

Archival formalin-fixed, paraffin-embedded (FFPE) tumor specimens were collected from advanced NSCLC patients enrolled in LETS phase III trial comparing first-line S-1/carboplatin with paclitaxel/carboplatin and subjected to multiplex genotyping for 214 somatic hotspot mutations in 26 genes (LungCarta Panel) and 20 major variants of *ALK*, *RET*, and *ROS1* fusion genes (LungFusion Panel) with the Sequenom MassARRAY platform. *MET* amplification was evaluated by fluorescence in situ hybridization. A somatic mutation in at least one gene was identified in 48% of non–squamous cell carcinoma and 45% of squamous cell carcinoma specimens, with *EGFR* (17%), *TP53* (11%), *STK11* (9.8%), *MET* (7.6%), and *KRAS* (6.2%). Mutations in *EGFR* or *KRAS* were associated with a longer or shorter median overall survival, respectively. The LungFusion Panel identified *ALK* fusions in six cases (2.5%), *ROS1* fusions in five cases (2.1%), and a *RET* fusion in one case (0.4%), with these three types of rearrangement being mutually exclusive. Nine (3.9%) of 229 patients were found to be positive for de novo *MET* amplification. This first multiplex genotyping of NSCLC associated with a phase III trial shows that MassARRAY-based genetic testing for somatic mutations and fusion genes performs well with nucleic acid derived from FFPE specimens of NSCLC tissue.

## INTRODUCTION

Lung cancer is the leading cause of death related to cancer worldwide,with non–small cell lung cancer (NSCLC) accounting for 85% of lung cancer cases (1). Advanced or metastatic NSCLC has been treated with platinum-based chemotherapies in a manner dependent on tumor histological features, with consideration given to the balance between the modest efficacy and side effects of such treatment. Over the last decade, however, substantial progress has been made in the development of genotype-based targeted therapies for advanced NSCLC. The success of epidermal growth factor receptor (EGFR) tyrosine kinase inhibitors (TKIs) in the treatment of *EGFR* mutation–positive advanced NSCLC established a proof of concept that molecularly targeted agents are far more effective than conventional chemotherapy when administered to the appropriate genetically defined patient population (2-7). Somatic mutations in other genes including *KRAS*, *HER2*, *PIK3CA*, *BRAF*, and *DDR2* have also been investigated as potential targets for genotype-based treatment approaches in NSCLC (8). More recently, the anaplastic lymphoma kinase (ALK) TKI crizotinib was approved with a companion diagnostic test for the treatment of a relatively small (up to 3 to 5%) subset of patients with advanced NSCLC who harbor *ALK* rearrangements (9-11). The subsequent discovery of *ROS1* and *RET* rearrangements as potentially treatable targets suggested that several chromosomal translocations and corresponding gene fusions may serve as a driving force for NSCLC (12-16). These findings have highlighted the genetic diversity of NSCLC, which can no longer be considered a single disease. Furthermore, the coexistence of different genetic alterations and therapeutic targets in NSCLC patients can profoundly affect the response to therapy (17). The clinical implementation of genomic profiling for NSCLC with high-throughput and multiplex genotyping tests is thus warranted in order to prioritize appropriate therapies for individual patients (18).

We have previously presented the results of the Lung Cancer Evaluation of TS-1 (LETS) study (19, 20). This multicenter randomized phase III trial demonstrated the noninferiority of the combination of S-1 and carboplatin compared with that of paclitaxel and carboplatin in terms of overall survival (OS) for chemotherapy-naïve patients with advanced NSCLC. Our West Japan Oncology Group (WJOG) has now embarked on multiplex genomic analyses of the archival formalin-fixed, paraffin-embedded (FFPE) tumor specimens collected from the patients enrolled in the LETS study. The primary platform for genotyping of tumors adopted in the present study is the Sequenom MassARRAY system, which combines multiplex polymerase chain reaction (PCR) analysis with single-base primer extension, followed by analysis of the primer extension products by matrix-assisted laser desorption-ionization (MALDI)–time-of-flight (TOF) mass spectrometry. We thus conducted high-throughput genotyping of 214 somatic hotspot mutations in 26 genes (LungCarta Panel) (Supplementary Table S1) as well as of 20 major variants of *ALK*, *RET*, and *ROS1* fusion genes (LungFusion Panel). Given that recent preclinical and clinical studies have also implicated de novo *MET* amplification as an oncogenic driver (21-23), we also evaluated *MET* amplification in available tumor specimens by fluorescence in situ hybridization (FISH).

## RESULTS

### Patients and sample collection

**Figure 1:**
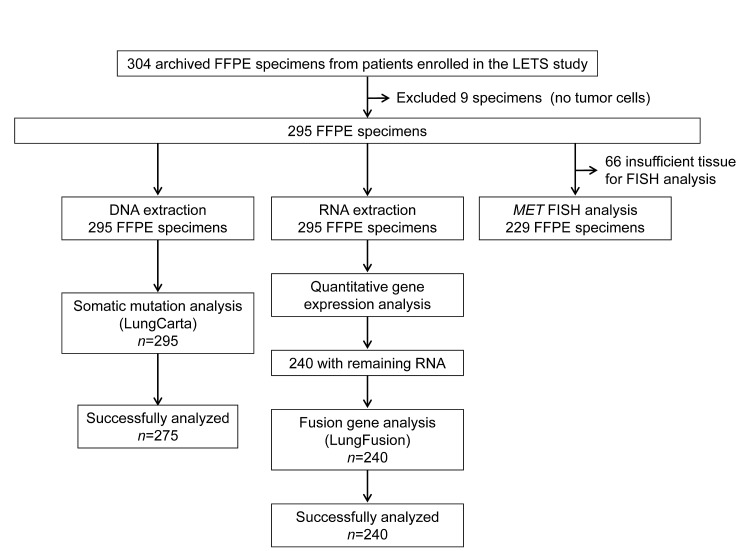
CONSORT diagram for the study Of the FFPE specimens obtained from 304 advanced NSCLC patients (54%) enrolled in the LETS study, 9 specimens contained no tumor cells and the remaining 295 specimens were subjected to extraction of DNA and RNA. In addition, 229 FFPE specimens were analyzed for *MET* amplification by FISH.

FFPE specimens obtained at diagnosis were available for 304 (53.9%) of the 564 patients enrolled in the LETS study. Most (229 out of 304, 75.3%) of the specimens were obtained by transbronchial biopsy. Nine specimens contained no tumor cells and were excluded from further analysis. The remaining 295 specimens were subjected to extraction of DNA and RNA, yielding median amounts of 504 ng (range, 33 to 25,230 ng) and 516 ng (range, 6 to 32,795 ng), respectively. The numbers of evaluable patients were 275 for somatic gene mutations (LungCarta Panel), 240 for fusion gene characterization (LungFusion Panel), and 229 for *MET* amplification (FISH) (Figure 1). The characteristics of these groups of patients, including the efficacy results, were similar overall to those of the intention-to-treat population (Table 1).

**Table 1:** Characteritics and outcome for patients subjected to molecular analyses compared with those for the intention-to-treat (ITT) population of the LETS study

	Somatic mutation analysis (*n* = 275)	Fusion gene analysis (*n* = 240)	*MET* amplification analysis (*n* = 229)	ITT population (*n* = 564)
*Characteristic*				
CBDCA+PTX/CBDCA+S-1	136 (49%)/139 (51%)	117 (49%)/123 (51%)	113 (49%)/116 (51%)	282 (50%)/282 (50%)
Median age (range), years	63 (36–74)	64 (36–74)	63 (36–74)	64 (36–74)
Male/female	211 (77%)/64 (23%)	184 (77%)/56 (23%)	178 (78%)/51 (22%)	433 (77%)/131 (23%)
ECOG PS 0/1	76 (28%)/199 (72%)	63 (26%)/177 (74%)	62 (27%)/167 (73%)	177 (31%)/387 (69%)
Clinical stage IIIB/IV	68 (25%)/207 (75%)	59 (25%)/181 (75%)	60 (26%)/169 (74%)	136 (24%)/428 (76%)
Nonsmoker/smoker	49 (18%)/226 (82%)	44 (18%)/196 (82%)	38 (17%)/191 (83%)	104 (18%)/460 (82%)
*Outcome*				
PFS hazard ratio (95% CI)	0.88 (0.70–1.12)	0.95 (0.74–1.24)	0.83 (0.64–1.09)	1.04 (0.86–1.22)
OS hazard ratio (95% CI)	0.93 (0.71–1.21)	0.85 (0.64–1.13)	0.91 (0.68–1.21)	0.96 (0.79–1.15)

Abbreviations: CBDCA, carboplatin; PTX, paclitaxel; ECOG, Eastern Cooperative Oncology Group; PS, performance status; PFS, progression-free survival; CI, confidence interval; OS, overall survival.

### Analysis of somatic gene mutations

**Figure 2:**
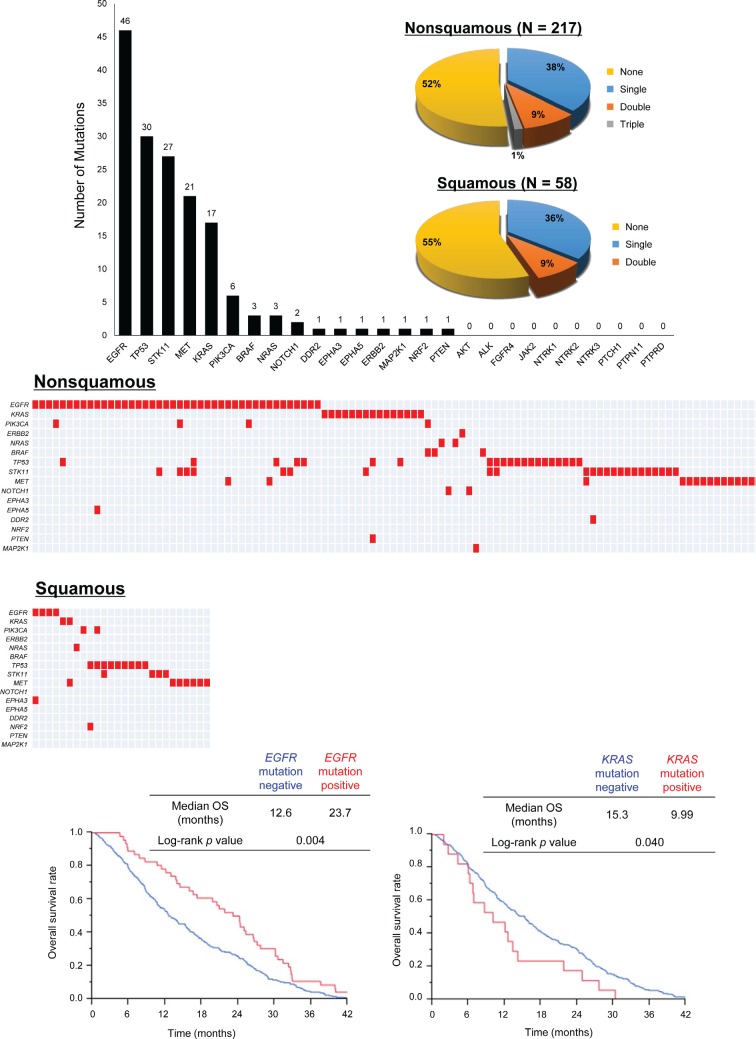
Analysis of somatic gene mutations in FFPE specimens from advanced NSCLC patients A, The pie charts show the distribution for the number of mutations detected in specimens according to tumor histology. B, Number of mutations in each of the 26 analyzed genes for the 275 specimens that were successfully genotyped. C, Mutational profiles for the patients harboring at least one mutation. D, OS analysis for advanced NSCLC patients according to *EGFR* mutation and *KRAS* mutation status.

Of the 295 specimens referred for somatic mutation analysis, 275 (93.2%) provided mutational profiles with a >90% success rate for genotyping (Figure 1). Somatic mutations in at least one gene were identified in 105 (48%) of the 217 patients with non–squamous cell carcinoma (non-SCC) and in 26 (45%) of the 58 patients with SCC. Twenty-five (9.1%) specimens (20 non-SCC, 5 SCC) were positive for mutations in two genes, and three non-SCC tumors each had mutations in three genes (Figure 2A). Overall, we identified *EGFR* mutations in 46 patients (17%), *TP53* mutations in 30 (11%), *STK11* mutations in 27 (9.8%), *MET* mutations in 21 (7.6%), *KRAS* mutations in 17 (6.2%), *PIK3CA* mutations in 6 (2.2%), *BRAF* and *NRAS* mutations in 3 each (1.1%), *NOTCH1* mutations in 2 (0.7%), and *DDR2*, *EPHA3*, *EPHA5*, *ERBB2*, *MAP2K1*, *NRF2*, and *PTEN* mutations in 1 each (0.4%) (Figure 2B). Among the 46 patients with *EGFR* mutations, 15 individuals (33%) had a deletion in exon 19 and 24 individuals (52%) had a point mutation (L858R or L861Q) in exon 21, whereas three patients had point mutations in exon 18, two had point mutations in exon 19, and two had mutations in exon 20 (Supplementary Table S2). Mutation profiles for patients harboring at least one mutation are shown in Figure 2C. *EGFR* and *KRAS* mutations were mutually exclusive. Of the 46 patients with *EGFR* mutations, three also harbored *PIK3CA* mutations. Four patients with *KRAS* mutations also had an additional mutation in *STK11*, in *TP53* and *PTEN*, in *TP53*, or in *MET*.

The median OS of *EGFR* mutation–positive patients was significantly longer than that of patients without *EGFR* mutations (23.7 vs. 12.6 months, *P* = 0.004) (Figure 2D). Conversely, patients with *KRAS* mutations had a significantly shorter median OS than did those with wild-type *KRAS* (9.99 vs. 15.3 months, *P* = 0.040) (Figure 2D).

### Fusion gene characterization

We previously established an assay system based on the MassARRAY platform for detecting *EML4-ALK* in FFPE biopsy specimens of advanced NSCLC (24). In the present study, we further developed a new multiplex system for MassARRAY assays (LungFusion Panel) focused on the capture of 20 major variants of *ALK*, *RET*, and *ROS1* fusion genes (Supplementary Tables S3 to S5). The LungFusion Panel assays detected plasmid DNA corresponding to the 20 different fusion variants with the expected mass spectra (Supplementary Figure S1), with the lower threshold for detection ranging from 5 to 60 copies (Supplementary Table S6).

**Figure 3:**
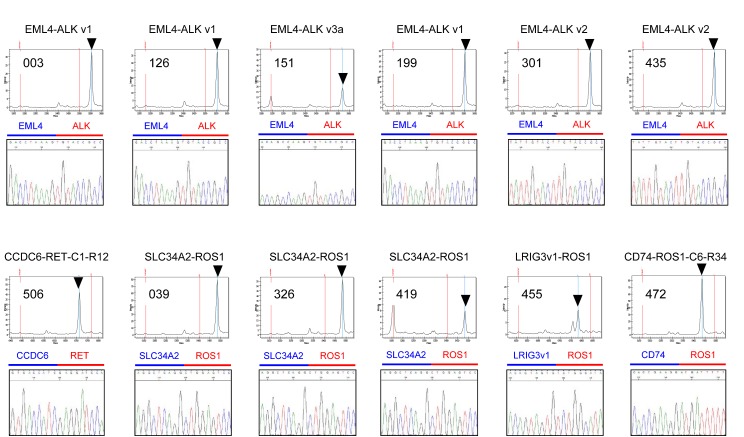
Detection of *ALK*, *RET*, and *ROS1* fusion genes in FFPE specimens of advanced NSCLC Arrowheads indicate mass spectrometry peaks corresponding to the indicated fusion genes. The variants of these fusions identified with the LungFusion Panel were validated by direct sequencing.

All 240 specimens referred for analysis with the LungFusion Panel were tested successfully. The LungFusion assay followed by direct sequencing identified *ALK* fusions in six cases (three *EML4-ALK* variant 1, two *EML4-ALK* variant 2, and one *EML4-ALK* variant 3a), a *CCDC6-RET* fusion in one case, and *ROS1* fusions in five cases (three *SLC34A2-ROS1*, one *LRIG3v1-ROS1*, and one *CD74-ROS1*) (Figure 3). The frequencies of *ALK*, *RET*, and *ROS1* rearrangements were 2.5%, 0.4%, and 2.1%, respectively, and these three types of rearrangement were mutually exclusive. Clinicopathologic characteristics of the 12 fusion-positive patients are shown in Table 2. Although these patients tended to be younger than the fusion-negative patients (median age of 58 vs. 64 years), there was no statistically significant difference in age, sex distribution, smoking history, tumor histological type, or disease stage between these two groups. Among the *ALK* fusion–positive patients, two individuals had concurrent *STK11* (F354L) mutations and one had a *MET* (N375S) mutation (Table 2). Among the five *ROS1* fusion–positive patients, two individuals also had a *KRAS* mutation (G12V or G12A) and one had *EGFR* (L858R), *PIK3CA* (E542K), and *STK11* (F354L) mutations (Table 2). The median OS was 19.5 and 13.8 months (*P* = 0.89) for fusion-positive and fusion-negative patients, respectively.

**Table 2:** Clinicopathologic characteristics of the 12 patients with fusion gene–positive NSCLC

Fusion gene	Age (years)	Sex	Smoking history	Tumor histology	Clinical stage	Concomitant mutations
*EML4-ALK v1*	70	F	No	Ad	IV	*STK11* (F354L)
*EML4-ALK v1*	50	M	Yes	Ad	IV	*MET* (N375S)
*EML4-ALK v3a*	55	M	Yes	Sq	IIIB	None
*EML4-ALK v1*	56	M	Yes	Ad	IV	None
*EML4-ALK v2*	57	F	No	Sq	IIIB	None
*EML4-ALK v2*	50	F	Yes	Ad	IIIB	*STK11* (F354L)
*CCDC6-RET*	58	F	No	Ad	IV	None
*SLC34A2-ROS1*	74	M	Yes	Ad	IV	*KRAS* (G12V)
*SLC34A2-ROS1*	65	F	No	Ad	IV	*EGFR* (L858R), *PIK3CA* (E542K), *STK11*(F354L)
*SLC34A2-ROS1*	58	M	Yes	Ad	IV	*KRAS* (G12A)
*LRIG3v1-ROS1*	65	M	Yes	Other	IV	None
*CD74-ROS1*	53	M	Yes	Ad	IIIB	None

Ad: Adenocarcinoma, Sq: Squamous cell carcinoma

### *MET* amplification

**Figure 4:**
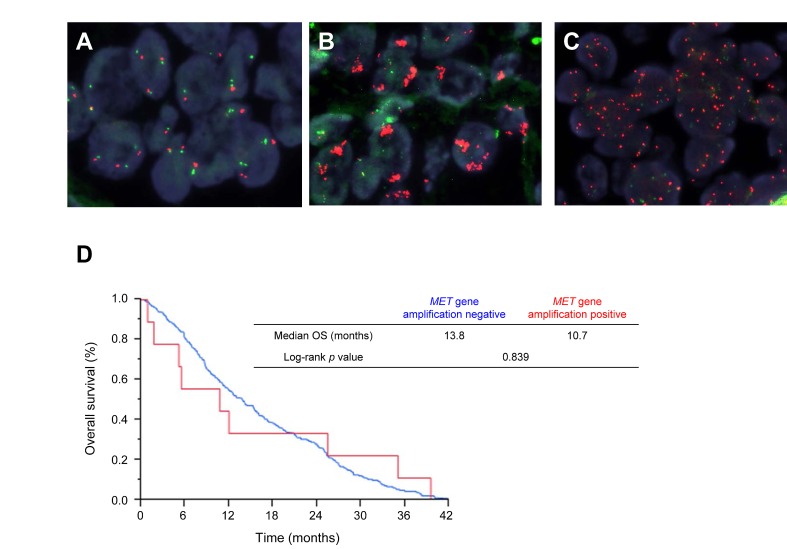
FISH analysis of de novo *MET* amplification in advanced NSCLC and survival analysis according to *MET* amplification status A–C, Representative FISH images for specimens negative (A) or positive (B and C) for *MET* amplification. Green and red signals correspond to CEN7p and the *MET* locus, respectively. D, OS according to de novo *MET* amplification status in advanced NSCLC patients.

*MET* copy number was evaluated by FISH in 229 cases and was detected in 9 cases (3.9%) (Figure 4A–C), among which the median gene copy number was 8.8 (range, 6.1 to 15.3). All *MET* amplification–positive patients had non-SCC (5.2%, 9 of 174 patients) and most were male and smokers (Table 3). Two of these patients had a *TP53* mutation, either alone or together with an *STK11* mutation, and one patient had two *EGFR* mutations (E709A + G719S) (Table 3). Although the median OS tended to be shorter for *MET* amplification–positive patients than for amplification-negative patients (10.7 vs. 13.8 months), this difference was not statistically significant (Figure 4D).

**Table 3:** Clinicopathologic characteristics of the nine patients with MET amplification–positive NSCLC

Age (years)	Sex	Smoking history	Tumor histology	Clinical stage	Concomitant mutations
54	M	Yes	Ad	IV	None
71	F	No	Ad-sq	IV	*TP53* (R248Q), *STK11* (F354L)
54	M	Yes	Ad	IV	*TP53* (R273L)
57	M	Yes	Ad	IV	None
59	M	No	Ad	IV	*EGFR* (E709A, G719S)
64	M	Yes	Ad	IV	None
46	M	Yes	Ad	IV	None
54	M	Yes	Ad	IV	None
72	M	Yes	Ad	IV	None

## DISCUSSION

As the number of molecularly targeted therapies for molecularly defined subsets of patients with NSCLC increases, there is an increasing need for high-throughput genotyping tests to evaluate the corresponding genetic abnormalities. The successful clinical application of such tests will depend on attainment of robust performance with minute samples derived from the FFPE tumor material collected for pathological diagnosis. In the present study, we tested FFPE specimens of NSCLC tissue for multiple genetic abnormalities simultaneously with the use of multiplex assay panels based on Sequenom's MassARRAY platform. The LungCarta Panel encompasses 214 distinct mutations in 26 genes previously annotated in NSCLC. Although collection of tumor material was not mandatory in the LETS study, FFPE archival tumor specimens were obtained from more than half of the advanced NSCLC patients enrolled in the study. Although most of the collected specimens were obtained by transbronchial biopsy and were small in size, >90% were successfully genotyped, thus satisfying the dual requirements of pathological diagnosis and multiplex analysis of somatic mutations with a single biopsy sample. We detected mutations in at least one gene in about half of the tested subjects, consistent with previous studies performed with other platforms (25). The frequency of *EGFR* mutations in our study (17%) is lower than that previously determined for Japanese patients with NSCLC (26). Given that *EGFR* mutation tests have been commercially available with insurance coverage since 2007 in Japan, the reason for this difference is likely that many *EGFR* mutation–positive patients were not enrolled in the LETS study because EGFR-TKIs were available as a first-line treatment option. The bias toward a higher percentage of wild-type *EGFR* patients may also have affected the observed incidence of other somatic mutations, including both those that are nonoverlapping or associated with *EGFR* mutations. The 6% prevalence of *KRAS* mutations in our cohort is also lower than the frequency reported for Caucasian patients, consistent with the previously described ethnic differences in the incidence of *KRAS* mutations (26). We also retrospectively evaluated the influence of *EGFR* or *KRAS* genotype on survival outcome for the advanced NSCLC patients enrolled in the LETS study. *EGFR* mutation–positive patients had a significantly superior OS compared with individuals with wild-type *EGFR*, likely because most mutation-positive patients received EGFR-TKIs as second-line or later chemotherapy. On the other hand, patients who had tumors with wild-type *KRAS* had a significantly better survival compared with those who had *KRAS* mutations. Given that some patients with wild-type *KRAS* had *EGFR* mutations or *ALK*, *RET*, or *ROS1* fusion genes, however, we also compared the survival outcome of *KRAS* mutation–positive patients with that of wild-type *KRAS* patients negative for these treatable targets. Although *KRAS* mutation–positive patients showed a trend toward a shorter survival compared with those negative for *KRAS* and *EGFR* mutations as well as for fusion genes (9.99 vs. 12.9 months, *P* = 0.113) (Supplementary Figure S2), the negative prognostic value of *KRAS* mutations remains uncertain on the basis of the data in the present study.

Several oncogenic gene fusions have recently been identified in NSCLC. *EML4-ALK* was the first such fusion detected in NSCLC, with its discovery in 2007 (9) being followed by the identification of *ROS1* and *RET* fusions in 2012 (12-15). Although the frequency of each of these types of fusion gene is only ~1 to 5% in unselected NSCLC patients, the affected patient subsets are treatable with corresponding kinase inhibitors. A break-apart FISH assay is the FDA-approved diagnostic test to screen for *ALK* rearrangement in NSCLC. FISH is thus currently considered the standard diagnostic technology for gene rearrangement, but its high cost and requirement for technical expertise limit its clinical application. Furthermore, timely acquisition of genotype information including oncogenic gene fusion status is required to guide rapid initiation of appropriate molecularly targeted therapies. The development of novel platforms that allow simultaneous screening for *ALK*, *ROS1*, and *RET* fusions is thus urgently needed. In the present study, we extended the MassARRAY technique to develop a multiplex screen (LungFusion Panel) designed to assess RNA isolated from FFPE biopsy specimens for *ALK*, *ROS1*, and *RET* fusion genes simultaneously. In this initial proof-of-concept effort, we confirmed robust performance of the LungFusion assay with 240 FFPE clinical samples obtained from advanced NSCLC patients, revealing a prevalence of 2.5%, 2.1%, and 0.4% for *ALK*, *ROS1*, and *RET* fusion genes, respectively. We also confirmed the mutual exclusivity of these three types of fusion gene. Of note, we found that three of five *ROS1* fusion–positive patients harbored concurrent actionable oncogenic somatic mutations of *EGFR*, *PIK3CA*, or *KRAS*. A 65-year-old woman who had never smoked had adenocarcinoma harboring *SLC34A2-ROS1* as well as *EGFR* (L858R) and *PIK3CA* (E542K) mutations. Two previous studies of Asian populations also detected coexistence of *EGFR* mutations and *ROS1* rearrangements in NSCLC patients (27, 28). Given that our cohort was also exclusively Japanese, the high prevalence of *EGFR* mutations in Asian patients with NSCLC may increase the chance for detection of coexistence of these two types of genetic alterations. As far as we are aware, the above-mentioned 65-year-old woman in our cohort is the first reported patient with both a *ROS1* fusion and a *PIK3CA* mutation. We also detected *KRAS* mutations (G12V or G12A) in two *SLC34A2-ROS1*–positive patients, with coexistence of *ROS1* rearrangement and *KRAS* mutation not having been previously described. Further studies are warranted to investigate whether the overlap between these oncogenes is clinically relevant and might affect the choice of optimal therapy.

We have previously shown that inhibition of MET signaling either with the small-molecule MET and ALK inhibitor crizotinib or by RNA interference targeted to MET mRNA resulted in marked antitumor effects in *MET* amplification–positive NSCLC cell lines both in vitro and in vivo (21). Furthermore, NSCLC patients with de novo *MET* amplification have shown a pronounced clinical response to crizotinib (22, 23), which was originally developed as a TKI for c-MET. These preclinical and clinical findings suggest that de novo *MET* amplification is an oncogenic driver for, and therefore a valid target for the treatment of, NSCLC. The clinicopathologic profile of advanced NSCLC patients with de novo *MET* amplification remains largely unknown, however. Several studies performed with different methods and different criteria for definition of gene amplification have reported a frequency of de novo *MET* amplification in NSCLC ranging from 2% to 20% (29). In the present study, we applied strict guidelines of the American Society of Clinical Oncology/College of American Pathologists for the definition of gene amplification and thereby identified 9 out of 229 advanced NSCLC patients (3.9%) as having de novo *MET* amplification. Eight of these nine patients had adenocarcinoma and one had adenosquamous carcinoma histology. Although most of the nine patients were male and smokers, no specific clinicopathologic feature was significantly associated with de novo *MET* amplification. The notion that tumors positive for de novo *MET* amplification, *EGFR* mutations, or oncogenic (*ALK*, *ROS1*, *RET*) fusions are distinct biological entities was supported by our finding that, with one exception, these genetic alterations were mutually exclusive.

There are several potential limitations to our study. First, although we detected significant survival differences between advanced NSCLC patients positive or negative for *EGFR* or *KRAS* mutations, the analysis did not take into account other prognostic factors and should be interpreted within the context of its retrospective nature. Second, although the LungCarta Panel encompasses >200 mutations across 26 cancer genes, important gene mutations may be present outside of the selected hotspot regions. Given that the MassARRAY system involves multiple primer sets for both PCR amplification and primer extension, the addition of new mutations to existing panels is straightforward but still requires effort. Lastly, we performed molecular testing with a single biopsy specimen, which may not be representative of all sites within a tumor.

In summary, the present study constitutes the first multiplex genotyping analysis of patients with advanced NSCLC enrolled in a phase III clinical trial. Such an approach will be important for future evaluation of the clinical impact of specific genetic alterations and predictive biomarkers. Our data indicate that MassARRAY-based multiplex genetic testing both for somatic mutations and for *ALK*, *ROS1*, and *RET* fusion genes performed well with nucleic acid (DNA and RNA) extracted from FFPE tumor specimens obtained from patients with advanced NSCLC.

## METHODS

### Patients and sample collection

The design and results of the LETS study have been described previously [19,20]. In brief, the study subjects comprised patients aged 20 to 74 years with a histopathologic diagnosis of stage IIIB or IV NSCLC, an Eastern Cooperative Oncology Group (ECOG) performance status of 0 or 1, and preserved function of major organ systems. They had not previously received chemotherapy, and they were randomly assigned in a 1:1 ratio to treatment with either carboplatin plus S-1 or carboplatin plus paclitaxel. The present study was designed retrospectively after completion of the first interim analysis of the LETS trial and was approved by the institutional ethics committee of each of the participating institutions. Archival FFPE tumor specimens were collected for diagnosis from the participants of the LETS study at 22 centers and were shipped to Kinki University Faculty of Medicine.

### Sample processing

The collected FFPE specimens underwent histological review, and only those containing sufficient tumor cells as revealed by hematoxylin-eosin staining were subjected to nucleic acid extraction. DNA and RNA were purified with the use of an Allprep DNA/RNA FFPE Kit (Qiagen, Valencia, CA). The isolated RNA was subjected to reverse transcription with the use of a High Capacity cDNA Reverse Transcription Kit (Applied Biosystems, Foster City, CA). The DNA and RNA samples were analyzed in the following order of priority: (1) multiplex analysis of somatic gene mutations (LungCarta Panel; Sequenom, San Diego, CA), (2) quantitative analysis of gene expression (results to be described elsewhere), and (3) characterization of *ALK*, *ROS1*, and *RET* fusion genes (LungFusion Panel).

### Mutation detection by mass spectrometry

The genes in the LungCarta Panel are listed in Supplementary Table S1. Multiplex PCR was performed in a volume of 5 μL containing 1 U of Hotstart Taq polymerase (Sequenom), 1.1 to 10 ng of genomic DNA, the LungCarta PCR primer pool(Sequenom), and 500 μmol of each deoxynucleoside triphosphate (dNTP). The PCR protocol included incubation at 95°C for 15 min; 45 cycles of incubation at 94°C for 20 s, 56°C for 30 s, and 72°C for 60 s; and a final incubation at 72°C for 3 min. Unincorporated dNTPs were deactivated by incubation with 0.5 U of shrimp alkaline phosphatase (Sequenom) at 37°C for 40 min, after which the enzyme was inactivated by incubation for 5 min at 85°C. Single-base primer extension was performed with the LungCarta extension primer pool (Sequenom), 0.2 μL of mass-modified dNTPs (Sequenom), and 1.15 U of Thermosequenase enzyme (Sequenom). The extension protocol included incubation at 94°C for 30 s; 60 cycles of incubation at 94°C for 5 s, 52°C for 5 s, and 80°C for 5 s; and a final incubation at 72°C for 3 min. After the addition of a cation-exchange resin to remove residual salt followed by 41 μL of water, the extension products were spotted onto a matrix pad (3-hydroxypicolinic acid) of a SpectroCHIP II (Sequenom) for analysis with a Bruker MALDI-TOF mass spectrometer. Spectra were processed with SpectroREADER software (Sequenom) and transferred to the MassARRAY Typer 4 Analyzer (Sequenom) for further analysis.

### Fusion gene detection by mass spectrometry

PCR and extension primers were designed to specifically amplify the breakpoint junction regions for 20 types of fusion gene (Supplementary Tables S3–S5) with the use of MassARRAY Assay Designer 3.1 (Sequenom). The detection technique has been described previously.^25^ Reverse-transcribed cDNA was subjected to PCR in a volume of 5 μL containing 1 U of Taq polymerase (Sequenom), 500 μmol of each dNTP, and 200 nmol of each PCR primer. The PCR protocol included incubation at 95°C for 15 min; 45 cycles of incubation at 94°C for 20 s, 56°C for 30 s, and 72°C for 60 s; and a final incubation at 72°C for 3 min. Unincorporated dNTPs were deactivated by incubation with 0.5 U of shrimp alkaline phosphatase (Sequenom) at 37°C for 40 min, after which the enzyme was inactivated by incubation for 5 min at 85°C. Single-base primer extension was performed with the LungFusion extension primer pool (depending on the mass), 0.2 μL of mass-modified dNTPs (Sequenom), and 1 U of iPLEX enzyme (Sequenom). The extension protocol included incubation at 94°C for 30 s; 40 cycles of incubation at 94°C for 5 s, 52°C for 5 s, and 80°C for 5 s; and a final incubation at 72°C for 3 min. After the addition of a cation-exchange resin to remove residual salt followed by 41 μL of water, the extension products were spotted onto a matrix pad (3-hydroxypicolinic acid) of a SpectroCHIP II (Sequenom) for analysis with a Bruker MALDI-TOF mass spectrometer. Spectra were processed with SpectroREADER software (Sequenom) and then transferred to the MassARRAY Typer 4 Analyzer (Sequenom) for further analysis.

Control vectors containing fusion sequences were constructed by In-Fusion PCR cloning (Clontech, Palo Alto, CA), with the exception of those for *EML4-ALK*, which were constructed as described previously [24]. Data analysis was performed with MassARRAY Typer software, version 4.0 (Sequenom). Positive samples were confirmed by subcloning and sequencing with the pTA2 vector (Toyobo, Osaka, Japan) and M13 universal primers.

### FISH

FISH was performed to determine *MET* copy number in FFPE tumor specimens with the use of a c-Met/CEN7p Dual Color FISH Probe (GSP Laboratory, Kawasaki, Japan), where CEN7p is the centromeric region of chromosome 7p. After screening of all sections, images of tumor cells were captured and recorded, and the signals for at least 50 random nuclei were counted for an area in which individual cells were recognized in each of at least 10 representative images. Nuclei with a disrupted boundary were excluded from the analysis. Gene amplification was strictly defined on the basis of a mean *MET*/CEN7p copy number ratio of >2.2, as previously described (30). Polysomy or an equivocal *MET*/CEN7p ratio (1.8 to 2.2) was thus scored as negative for amplification.

### Statistical analysis

OS in patients for each biomarker analysis was estimated with the Kaplan-Meier method and analyzed with a Cox proportional-hazard model. Differences in OS between genotypes were evaluated with the log-rank test. All statistical analysis was performed with SAS for Windows, release 9.2 (SAS Institute, Cary, NC), and JMP software (version 10, SAS Institute). A *P* value of <0.05 was considered statistically significant.

## SUPPLEMENTARY FIGURES AND TABLES


