# Supplementary material for: Multiplex genomic profiling of non–small cell lung cancers from the LETS phase III trial of first-line S-1/carboplatin versus paclitaxel/carboplatin: results of a West Japan Oncology Group study
Source: Oncotarget. 2014 Apr 17;5(8):2293–304. doi: 10.18632/oncotarget.1906 (PMC4039163; doi:10.18632/oncotarget.1906)
Supplement: Supplementary file 1 [file oncotarget-05-2293-s001.pdf]

Multiplex genomic profiling of non–small cell lung cancers from the LETS phase III trial of first-line S-1/carboplatin versus paclitaxel/carboplatin: results of a west Japan oncology group study

Supplementary Information

Supplementary Table S1: LungCarta Panel

Gene	Mutation detection
AKT1	E17K
ALK	C1156Y, L1196M
BRAF	D594G/V, G469S/E/A/V, L597Q/V, V600E/K/M
DDR2	C580Y, D125Y, G253C, G505S, G774E/V, I120M, I638F, L239R, L63V, T765P
EGFR	R108K, T263P, A289V, G598V, E709K/H, E709A/G/V, G719S/C/A/D/R/V, M766_A767insAI, D761Y/N, S768I, R776C/H, V769_D770insASV, V769_D770insCV, D770_N771>AGG/N771>GF, N771T, D770_N771insG, N771_P772>SVDNR, P772_H773insV, H773N, H773_V774insNPH/PH/H, V774L, V774_C775insHV, T790M, L858R/M, L861Q/R, E746_A750del, E746_T751del, E746_S752>V, E746_T751>A, E746_T751>V, E746_S752>A, E746_S752>D, L747_E749del, L747_A750del, L747_T751del, L747_P753>S, L747S, L747_S752del, A750P, T751A/P/I, S752F, S752_I759del, P753S/Q, E746_S752>I, E746_T751>A, L747_A750>P, L747_T751>P, L747_P753>Q, T854A, E746_T751>S, D770_N771insG, V769_D770insASV, D770_N771insGL, D770fs*61, D770_N771insAPW
EPHA3	A435S, D446Y, S449F, D806N, G187R, G518L, K761N, G766E, M269I, N379K, N85S, S229Y, T166N, T37K, T393K, W250R
EPHA5	D493Y, G582E, M1034I, N1032S, R1007Q, S566Y, S810I, T856I
ERBB2	M774_A775insAYVM, A775_G776insAYVM
FGFR4	P672T, H192fs*19
JAK2	L609S, P503L, R1122P, Y931C
KRAS	G12S/V/F/R/A/C/D, G13C/S/A/V/D, Q61L/R/P/H/E/K
MAP2K1	D67N, K57N, Q56P
STK11	A347fs*13, A43_L50del6, D327fs*10, E120*, E165*, E223*, E70*, E70fs*26, F354L, G163C, G188fs*99, G196V, G56fs*4, G56W, G91L, H174R, I26fs*25, K191*, K78E, L285Q, L50_D53del4, M51fs*14, P179L, Q123R, Q137*, Q159*, Q170*, Q220*, Q37L, R426W, R86G, V197fs*69, V236fs*30, Y272Y

MET	N375S, 982_1028del47
NOTCH1	H2276fs*79, D1643H, R2328W, T1997M, V1672I, V2444fs*35
NRAS	Q61E/K/H/L/R/P
NRF2	D29H, D77N/A, E79Q/K/G, G31A, G81D, R34Q
NTRK1	Q80*, R119H, S326R
NTRK2	Q666R, C45F, G261R, L138F, L670M, L755L
NTRK3	I769N, L152I, L248M, L270M, L336Q, S184C, T283K, V307L, R721G
PIK3CA	E542Q/K, E545Q/K, H1047Y/R/L
PTCH1	R1308G, R682L, S1326fs*46
PTEN	R233*
PTPN11	E76V
PTPRD	D1162N, D154Y, I44I, L1036Q, P1809R, R1536L, R584S, S1703R, T337A, V483E
TP53	G245C/S, G245D/V, R158C/G/L/P, R175L/H, R248G/L/Q/W, R249S/W/M, R273C/H/L/P, R282G/W, V157F, Y163C, R175L/H, Y220C

Supplementary Table S2: Type of EGFR Mutation in 46 EGFR mutation positive patients

exon	Type of mutation	Number of patients (n = 46)
exon18	G719A	2
	E709A/G719S*	1
exon19	T751I	1
	T751A	1
	E746_A750del	8
	E746_T751del, V ins	2
	L747_T751del	2
	L747_T750del, P ins	1
	L747_S752del, P753S	2
exon20	V769_D770insASV	1
	H773_V774insPH	1
exon21	L858R	22
	L861Q	2

*Double EGFR mutation was detected in one patients.

Supplementary Table S3: LungFusion Panel

ALK fusion genes	*RET* fusion genes	*ROS1* fusion genes
KIF5B-ALK-K17-A20	KIF5B-RETv2-K16-R12	CD74-ROS1-C6-R32
EML4-ALK v1	KIF5B-RETv4-K15-R12	CD74-ROS1-C6-R34
EML4-ALK v2	KIF5B-RETv4-K22-R12	SLC34A2-ROS1
EML4-ALK v3a	KIF5B-RETv4-K24-R11	SLC34A2-ROS-long
	CCDC6-RET-C1-R12	SLC34A2-ROS-short
		LRIG3v1-ROS1
		SDC4-ROS1-S2-R32
		SDC4-ROS1-S4-R32
		SDC4-ROS1-S4-R34
		EZR-ROS1-E10-R34
		TPM3-ROS1

Supplementary Table S4: Junction sequences of fusion cDNAs

Fusion gene	Junction sequence (5′-3′)
KIF5B-ALK-K17-A20	CCCTCAGTGAAGAACTAGTCCAGCTTCGAGCACAAG|TGTACCGCCGGAAGCACCAGGAGCTGCAAGCCATGCAGAT
EML4-ALK v1	TACTGTAGAGCCCACACCTGGGAAAGGACCTAAAG|TGTACCGCCGGAAGCACCAGGAGCTGCAAGCCATGCAGAT
EML4-ALK v2	TCTAACTCGGGAGACTATGAAATATTGTACT|TGTACCGCCGGAAGCACCAGGAGCTGCAAGCCATGCAGAT
EML4-ALK v3a	TACCAAAACTGCAGACAAGCATAAAGATGTCATCATCAACCAAG|TGTACCGCCGGAAGCACCAGGAGCTGCAAGCCATGCAG
KIF5B-RETv2-K16-R12	AGGAGTTAGCAGCATGTCAGCTTCGTATCTCTCAA|GAGGATCCAAAGTGGGAATTCCCTCGGAAGAACTTGGTTCTTGG
KIF5B-RETv4-K15-R12	CAGAAATAGGAATTGCTGTGGGAAATAATGATGTAAAG|GAGGATCCAAAGTGGGAATTCCCTCGGAAGAACTTGGTTCTTGGAAAAACTCTAGC
KIF5B-RETv4-K22-R12	TTTGTTCAGGACCTGGCTACAAGAGTTAAAAAG|GAGGATCCAAAGTGGGAATTCCCTCGGAAGAACTTGGTTCTTGG
KIF5B-RETv4-K24-R11	AGAATATGGCCAGAAGAGGGCATTCTGCACAGATTG|ATCCACTGTGCGACGAGCTGTGCCGCACGGTGATCGCAGCCGCTGTCCTCTTCTCCTT
CCDC6-RET-C1-R12	AAGGCACTGCAGGAGGAGAACCGCGACCTGCGCAAAGCCAGCGTGACCATC|GAGGATCCAAAGTGGGAATTCCCTCGGAAGAACTTGGTTCT
CD74-ROS1-C6-R32	TTGAAATGAGCAGGCACTCCTTGGAGCAAAAGCCCACTGACGCTCCACCGAAAG|CTGGAGTCCCAAATAAACCAGGC
CD74-ROS1-C6-R34	AAATGAGCAGGCACTCCTTGGAGCAAAAGCCCACTGACGCTCCACCGAAAG|ATGATTTTTGGATACCAGAAACAAGTTTC
SLC34A2-ROS1	CTCCTGAGACCTTTGATAACATAACCATTAGCAGAGAGGCTCAGG|CTGGAGTCCCAAATAAACCAGGCATTCCCAAATTAC
SLC34A2-ROS-long	ACTTTTTCGTGTGCTCCCTGGATATTCTTAGTAGCGCCTTCCAGCTGGTTGG|AGCTGGAGTCCCAAATAAACCAGGCATTCCCAAATTAC
SLC34A2-ROS-short	ACTTTTTCGTGTGCTCCCTGGATATTCTTAGTAGCGCCTTCCAGCTG|GTTGGAGATGATTTTTGGATACCAGAAACAAGTTTC
LRIG3v1-ROS1	CTTCAGGTGCTGGATTTTTCTTACCACAACATGACAGTAGTG|TCTGGCATAGAAGATTAAAGAATCAAAAAAGTGCCAAGGAAGG
SDC4-ROS1-S2-R32	GGAATCTGATGACTTTGAGCTGTCTGGCTCTGGAGATCTGG|CTGGAGTCCCAAATAAACCAGGCATTCCCAAATTAC
SDC4-ROS1-S4-R32	AGGGCAGCAACATCTTTGAGAGAACGGAGGTCCTGGCAG|CTGGAGTCCCAAATAAACCAGGCATTCCCAAATTAC
SDC4-ROS1-S4-R34	AGGGCAGCAACATCTTTGAGAGAACGGAGGTCCTGGCAG|ATGATTTTTGGATACCAGAAACAAGTTTCATAC
EZR-ROS1-E10-R34	GAAGGCAGAGAGAG|ATGATTTTTGGATACCAGAAACAAGTTTCATACTTACTATTATAGTTGGAATATTTCTGGTTGTTACAATCCCACT
TPM3-ROS1	GTAGCCAAGCTGGAAAAGACAATTGATGACCTGGAAG|TCTGGCATAGAAGATTAAAGAATCAAAAAAGTGCCAAGGAAGGGGTGACAGTGCTT

Supplementary Table S5: PCR (F, forward; R, reverse) and extension (EXT) primers for the LungFusion Panel

Fusion gene	Primer	Sequence	EXT mass	EXT call	Plex
KIF5B-ALK-K17-A20	F	5’- ACGTTGGATGATCTGCATGGCTTGCAGCTC -3’			
	R	5’- ACGTTGGATGCCCTCAGTGAAGAACTAGTC -3’			
	EXT	5’- CGGTACACTTGTGCTCG -3’	5448.6	T	1
EML4-ALK v1	F	5’- ACGTTGGATGATCTGCATGGCTTGCAGCTC -3’			
	R	5’- ACGTTGGATGTACTGTAGAGCCCACACCTG -3’			
	EXT	5’- CGGCGGTACACTTTAGG -3’	5553.5	A	9
EML4-ALK v2	F	5’- ACGTTGGATGATCTGCATGGCTTGCAGCTC -3’			
	R	5’- ACGTTGGATGATGTCTAACTCGGGAGACTA -3’			
	EXT	5’- CGGCGGTACAAGTACAA -3’	5555.5	A	10
EML4-ALK v3a	F	5’- ACGTTGGATGTACCAAAACTGCAGACAAGC -3’			
	R	5’- ACGTTGGATGATCTGCATGGCTTGCAGCTC -3’			
	EXT	5’- GCGGTACACTTGGTTGA -3’	5568.5	A	8
KIF5B-RETv2-K16-R12	F	5’- ACGTTGGATGAGGAGTTAGCAGCATGTCAG -3’			
	R	5’- ACGTTGGATGCCAAGAACCAAGTTCTTCCG -3’			
	EXT	5’- TATCTCTCAAGAGGATCCAAA -3’	6685.4	G	7
KIF5B-RETv4-K15-R12	F	5’- ACGTTGGATGTCTCCTAGAGTTTTTCCAAG -3’			
	R	5’- ACGTTGGATGCAGAAATAGGAATTGCTGTGG -3’			
	EXT	5’- TTTGGATCCTCCTTTACATCAT -3’	6953.4	A	5
KIF5B-RETv4-K22-R12	F	5’- ACGTTGGATGTTTGTTCAGGACCTGGCTAC -3’			

	R	5’- ACGTTGGATGCCAAGAACCAAGTTCTTCCG -3’			
	EXT	5’- AGAGTTAAAAAGGAGGATCCAAA -3’	7449.9	G	1
KIF5B-RETv4-K24-R11	F	5’- ACGTTGGATGATGAAGGAGAAGAGGACAGC -3’			
	R	5’- ACGTTGGATGAGAATATGGCCAGAAGAGGG -3’			
	EXT	5’- GTCGCACAGTGGATCAATC -3’	6139.9	A	4
CCDC6-RET-C1-R12	F	5’- ACGTTGGATGAAGGCACTGCAGGAGGAGAA -3’			
	R	5’- ACGTTGGATGAGAACCAAGTTCTTCCGAGG -3’			
	EXT	5’- AGCGTGACCATCGAGGATCCA -3’	6711.4	A	2
CD74-ROS1-C6-R32	F	5’- ACGTTGGATGGCCTGGTTTATTTGGGACTC -3’			
	R	5’- ACGTTGGATGTTGAAATGAGCAGGCACTCC -3’			
	EXT	5’- TGGGACTCCAGCTTTCG -3’	5464.6	C	6
CD74-ROS1-C6-R34	F	5’- ACGTTGGATGGAAACTTGTTTCTGGTATCC -3’			
	R	5’- ACGTTGGATGAAATGAGCAGGCACTCCTTG -3’			
	EXT	5’- CGCTCCACCGAAAGATG -3’	5435.6	A	4
SLC34A2-ROS1	F	5’- ACGTTGGATGGTAATTTGGGAATGCCTGGT -3’			
	R	5’- ACGTTGGATGCTCCTGAGACCTTTGATAAC -3’			
	EXT	5’- GGACTCCAGCCTGAGCC -3’	5483.5	A	3
SLC34A2-ROS-long	F	5’- ACGTTGGATGGTAATTTGGGAATGCCTGGT -3’			
	R	5’- ACGTTGGATGACTTTTTCGTGTGCTCCCTG -3’			
	EXT	5’- GGGACTCCAGCTCCAACCA -3’	6030.0	C	2
SLC34A2-ROS-short	F	5’- ACGTTGGATGACTTTTTCGTGTGCTCCCTG -3’			
	R	5’- ACGTTGGATGGAAACTTGTTTCTGGTATCC -3’			

	EXT	5’- CTTCCAGCTGGTTGGAGAT -3’	6122.0	G	3
LRIG3v1-ROS1	F	5’- ACGTTGGATGCTTCAGGTGCTGGATTTTTC -3’			
	R	5’- ACGTTGGATGCCTTCCTTGGCACTTTTTTG -3’			
	EXT	5’- GACAGTAGTGTCTGGCATAGA -3’	6781.5	A	1
SDC4-ROS1-S2-R32	F	5’- ACGTTGGATGGTAATTTGGGAATGCCTGGT -3’			
	R	5’- ACGTTGGATGGGAATCTGATGACTTTGAGC -3’			
	EXT	5’- CTCCAGCCAGATCTCCA -3’	5362.5	C	7
SDC4-ROS1-S4-R32	F	5’- ACGTTGGATGAGGGCAGCAACATCTTTGAG -3’			
	R	5’- ACGTTGGATGGTAATTTGGGAATGCCTGGT -3’			
	EXT	5’- AGGTCCTGGCAGCTGGAGT -3’	6132.0	C	5
SDC4-ROS1-S4-R34	F	5’- ACGTTGGATGAGGGCAGCAACATCTTTGAG -3’			
	R	5’- ACGTTGGATGGTATGAAACTTGTTTCTGG -3’			
	EXT	5’- GGTCCTGGCAGATGATTTT -3’	6176.9	T	1
EZR-ROS1-E10-R34	F	5’- ACGTTGGATGGAAGGCAGAGAGAGATGATT -3’			
	R	5’- ACGTTGGATGAGTGGGATTGTAACAACCAG -3’			
	EXT	5’- TTTGGATACCAGAAACAAG -3’	6171.9	T	6
TPM3-ROS1	F	5’- ACGTTGGATGATAAGCACTGTCACCCCTTC -3’			
	R	5’- ACGTTGGATGGTAGCCAAGCTGGAAAAGAC -3’			
	EXT	5’- TCTATGCCAGACTTCCA -3’	5392.5	C	2

Supplementary Table S6: Assay sensitivity for the LungFusion Panel

Fusion gene	Minimum number of copies detected
KIF5B-ALK-K17-A20	10
EML4-ALK v1	10
EML4-ALK v2	5
EML4-ALK v3a	5
KIF5B-RETv2-K16-R12	5
KIF5B-RETv4-K15-R12	30
KIF5B-RETv4-K22-R12	10
KIF5B-RETv4-K24-R11	5
CCDC6-RET-C1-R12	20
CD74-ROS1-C6-R32	60
CD74-ROS1-C6-R34	5
SLC34A2-ROS1	10
SLC34A2-ROS-long	5
SLC34A2-ROS-short	60
LRIG3v1-ROS1	20
SDC4-ROS1-S2-R32	10
SDC4-ROS1-S4-R32	10
SDC4-ROS1-S4-R34	30
EZR-ROS1-E10-R34	60
TPM3-ROS1	10

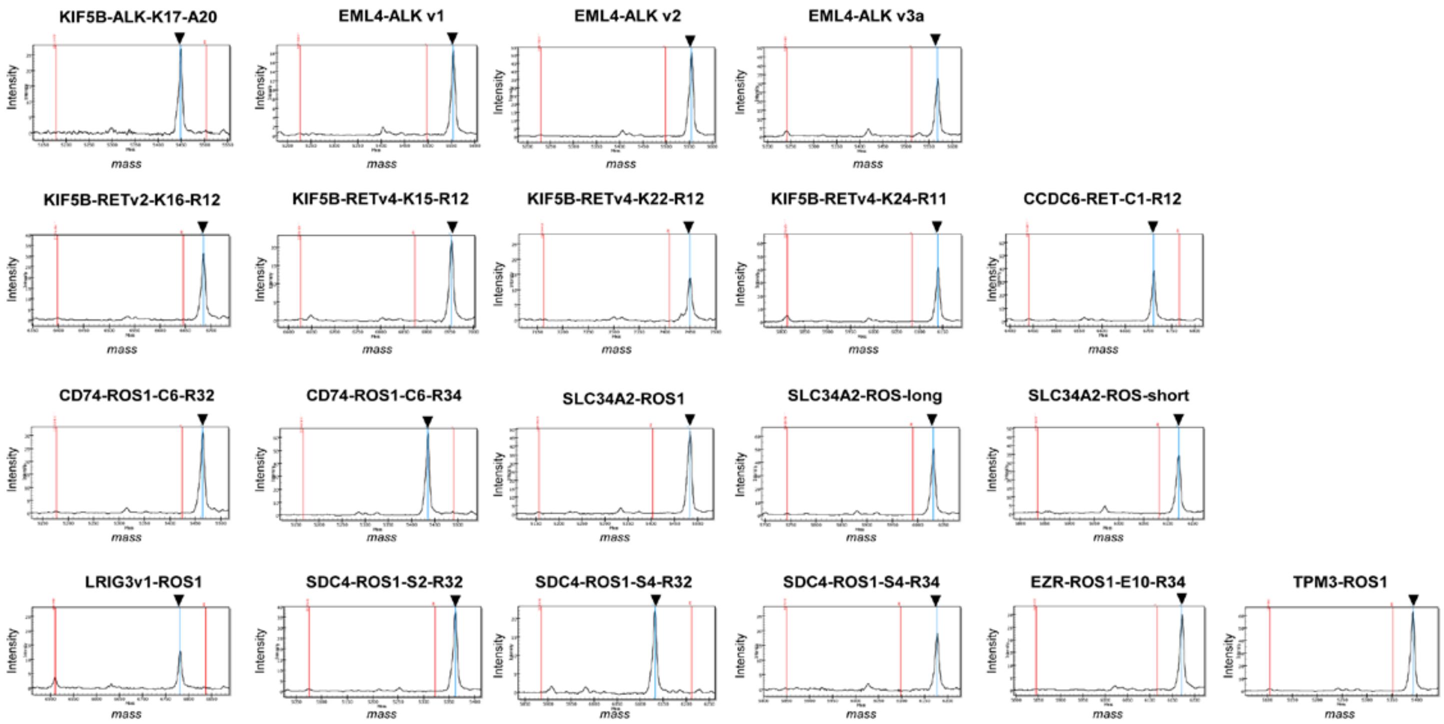

Supplementary Figure S1: Representative mass spectra for *ALK*, *RET*, and *ROS1* fusion variants. Arrowheads indicate mass spectrometry peaks corresponding to the indicated fusion genes.

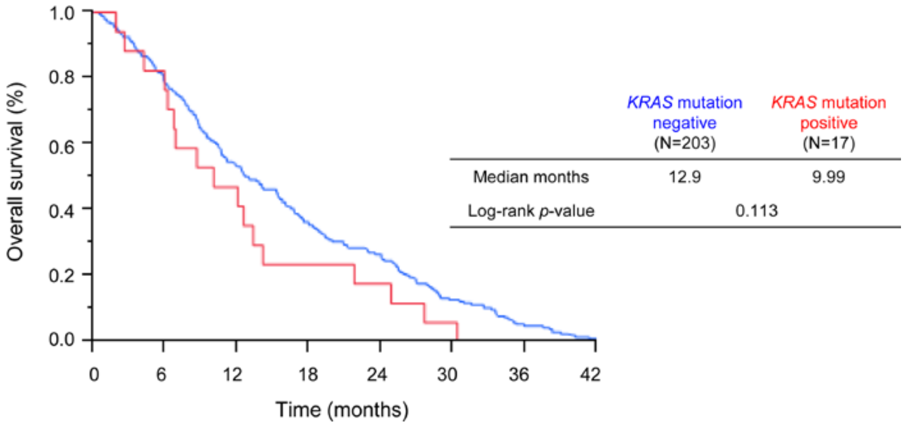

Supplementary Figure S2: OS analysis according to *KRAS* mutation status. *KRAS* mutation–positive patients (n = 17) were compared with patients negative for *KRAS* and *EGFR* mutations as well as for *ALK*, *RET*, and *ROS1* fusion genes (n = 203).
